# Acquired hemophilia A in an elderly female with bullous pemphigoid: Case report

**DOI:** 10.1097/MD.0000000000045325

**Published:** 2025-10-24

**Authors:** Zhongmei Liu, Junzhu Xu

**Affiliations:** a Department of Endocrinology and Hematology, Hangzhou Third People’s Hospital, Hangzhou City, Zhejiang Province, China; b Department of Dermatology, Hangzhou Third People’s Hospital, Hangzhou City, Zhejiang Province, China.

**Keywords:** acquired hemophilia, bullous pemphigoid, case report, elderly female, severe complications

## Abstract

**Rationale:**

Acquired hemophilia A (AHA) is a rare but life-threatening bleeding disorder, necessitating enhanced recognition in patients with concomitant autoimmune diseases.

**Patient concerns:**

A 91-year-old female with bullous pemphigoid presented with spontaneous subcutaneous bleeding and prolonged activated partial thromboplastin time.

**Diagnoses:**

Laboratory investigations revealed a decrease in factor VIII activity to 6.4%, confirming the diagnosis of bullous pemphigoid-associated AHA.

**Interventions:**

The patient was subjected to monotherapy with glucocorticoids and supportive hemostatic measures.

**Outcomes:**

Coagulation parameters normalized within 1 month, and no severe complications occurred.

**Lessons:**

This case highlights the importance of considering AHA in elderly patients with autoimmune diseases who present with unexplained bleeding. An individualized treatment strategy may improve clinical outcomes.

## 1. Introduction

Acquired hemophilia A (AHA) is a rare but serious bleeding disorder caused by the development of autoantibodies against coagulation factor VIII (FVIII).^[1]^ Unlike congenital hemophilia, which is linked to genetic mutations, AHA occurs in individuals with no prior bleeding history and typically presents with spontaneous or excessive bleeding in soft tissues, muscles, or mucous membranes.^[2,3]^ The condition is often diagnosed when patients present with unexplained prolonged activated partial thromboplastin time (APTT) that does not correct with standard mixing studies. Because of its rarity, AHA is frequently misdiagnosed, leading to delays in appropriate treatment and an increased risk of severe hemorrhagic complications.

AHA is most commonly associated with underlying conditions such as autoimmune diseases, malignancies, pregnancy, or infections, though in many cases, no identifiable cause is found.^[4,5]^ Among autoimmune conditions, bullous pemphigoid (BP), a chronic blistering skin disorder, has been reported in association with AHA, suggesting a possible immune-mediated link between the 2 diseases.^[6,7]^ The exact mechanism remains unclear, but it is believed that immune dysregulation in BP may trigger the production of FVIII autoantibodies, leading to secondary AHA. Given that both conditions predominantly affect the elderly, their coexistence presents diagnostic and therapeutic challenges due to increased vulnerability to complications and limited treatment options.

Here, we report a case of a 91-year-old woman with a history of BP who developed spontaneous bleeding and prolonged APTT, ultimately diagnosed as BP-related AHA. The case highlights the importance of early recognition of AHA in patients with autoimmune diseases, particularly those presenting with sudden-onset unexplained bleeding. We also emphasize the role of steroid monotherapy in frail elderly patients, demonstrating that aggressive immunosuppressive therapy may not always be necessary for disease control.

## 2. Case presentation

**Figure 1.**
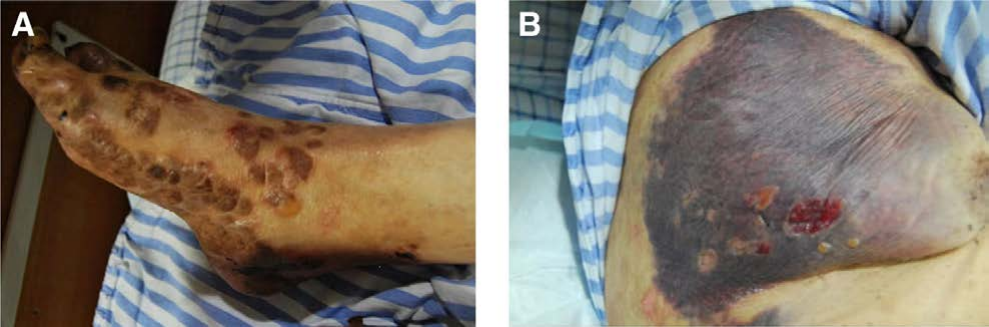
Representative images of subcutaneous bleeding.

**Figure 2.**
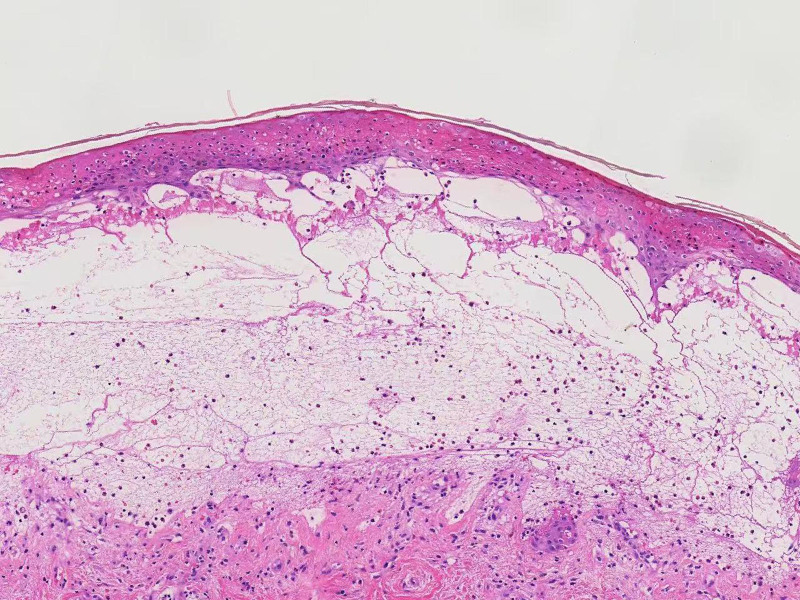
Histopathology of lesional skin showing a subepidermal blister with inflammatory cell infiltration, predominantly eosinophils (H&E stain, 200×).

**Figure 3.**
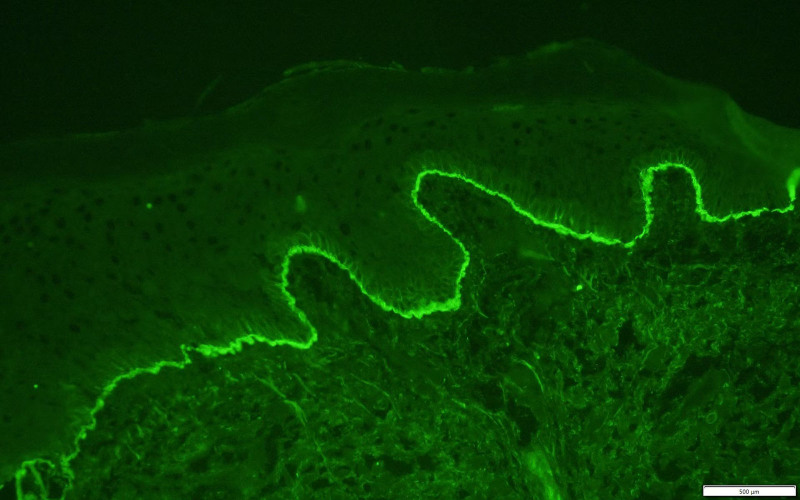
Direct immunofluorescence of perilesional skin showing linear IgG and C3 deposition along the basement membrane zone, diagnostic of bullous pemphigoid.

A 91-year-old female with a history of BP and schizophrenia presented to our hospital with diffuse skin blisters and spontaneous subcutaneous bleeding in the right axillary region (Fig. 1A). The baseline characteristics of the patient are summarized in Table 1. She had developed multiple bullous lesions on her limbs and trunk 2 months earlier and was diagnosed with BP based on clinical findings and serology: anti-BP180 strongly positive (167 U/mL), anti-desmoglein 1/3 negative (Dsg1 8 U/mL; Dsg3 5 U/ml). Skin biopsy from a bullous lesion showed subepidermal blister formation with prominent eosinophilic infiltration in the superficial dermis (Fig. 2). Direct immunofluorescence of perilesional skin demonstrated linear deposition of IgG and C3 along the dermo-epidermal junction (Fig. 3), consistent with the diagnosis of BP.

**Table 1:** Baseline characteristics of the patient.

Characteristic	Patient data
Age	91 yr
Sex	Female
Underlying conditions	Bullous pemphigoid, schizophrenia
Presenting symptoms	Subcutaneous bleeding, bullous skin lesions
Coagulation tests	APTT: 75.0 s, d-dimer: 1.81 mg/L, fibrinogen: 4.27 g/L
Factor VIII activity	6.4%
Factor VIII inhibitor titer	0 BU (normal: 0–0.6 BU)
Imaging findings	Right shoulder fluid collection (17.4 × 5.9 cm), interstitial lung disease with infection
Initial treatment	Plasma transfusion, cryoprecipitate, and methylprednisolone
Complications	Gastrointestinal bleeding (melena, hypotension, and anemia)
Final outcome	APTT normalization, FVIII activity 23.8%, no recurrent bleeding

APTT = activated partial thromboplastin time, FVIII = factor VIII.

Despite topical corticosteroid treatment, her condition remained unstable. Three days before admission, she developed spontaneous right axillary hematoma, prompting further evaluation (Fig. 1B). Laboratory tests revealed prolonged APTT (75.0 s), reduced FVIII activity (6.4%), and a normal FVIII inhibitor titer (0 BU), raising suspicion of AHA. She had no personal or family history of bleeding disorders and had previously undergone hip replacement surgery without abnormal bleeding. Imaging studies showed a 17.4 × 5.9 cm fluid collection in the right shoulder region, and chest CT revealed interstitial lung disease with infection. She was started on plasma transfusions, cryoprecipitate, and methylprednisolone (40 mg/d). During hospitalization, she developed gastrointestinal bleeding with melena, hypotension, and anemia, requiring activated prothrombin complex concentrate (aPCC), somatostatin, and red blood cell transfusions. Her bleeding was controlled, and APTT normalized within 20 days, with FVIII activity increasing to 23.8%. Due to her age and comorbidities, additional immunosuppressive therapy was avoided, and she was discharged on prednisone (15 mg/d), which was gradually tapered over 9 months. Follow-up showed no recurrence of hemorrhage or skin lesions, confirming a successful steroid-based treatment approach.

(A) Diffuse skin blisters and spontaneous subcutaneous bleeding in the right foot. (B) Spontaneous right axillary hematoma.

## 3. Treatment and outcome

**Figure 4.**
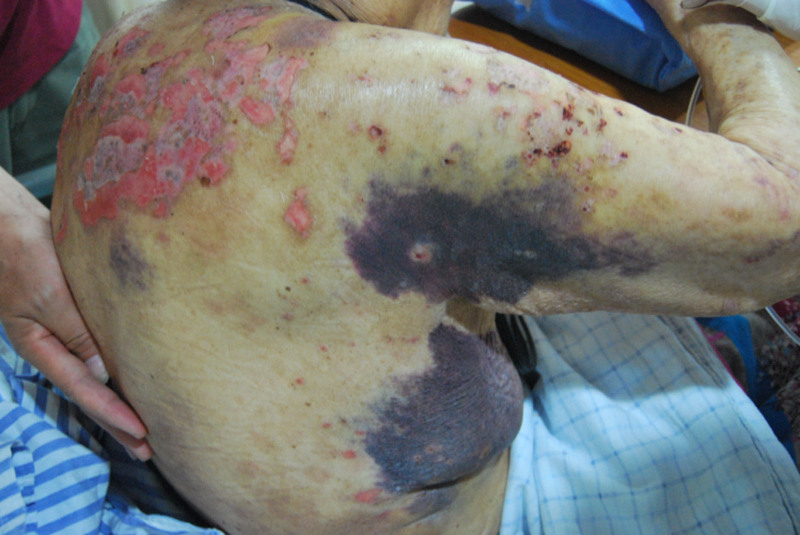
Representative image of resolved skin lesions and hematomas.

The patient was initially treated with plasma transfusion, cryoprecipitate (10 U), and intravenous methylprednisolone (40 mg/d) to control the bleeding and correct the coagulation abnormality. Over the first 5 days, her APTT began to shorten, and the subcutaneous hematoma stabilized without further progression. However, on the sixth day of hospitalization, she developed melena, hypotension, and tachycardia, indicating gastrointestinal hemorrhage, which required emergency intervention with somatostatin infusion, transfusion of red blood cells, and administration of aPCC (80 IU/kg/d). After 5 days of intensive hemostatic therapy, the gastrointestinal bleeding was controlled, and the coagulation parameters began to improve. Given her advanced age and the presence of interstitial lung disease with infection, additional immunosuppressive agents, such as cyclophosphamide or rituximab, were not initiated. Corticosteroids were gradually tapered over 2 weeks, and by day 20, her APTT had normalized, and FVIII activity had increased to 23.8%. At 1-month follow-up, she had no new hemorrhagic episodes, her skin lesions had resolved, and her hematomas had significantly regressed (Fig. 4). She was discharged on oral prednisone (15 mg/d), which was gradually tapered and discontinued after 9 months. Regular follow-ups confirmed the absence of recurrent bleeding or skin lesions, demonstrating the efficacy of corticosteroid monotherapy in controlling BP-associated AHA in this patient.

## 4. Discussion

AHA is a rare autoimmune disorder characterized by the development of autoantibodies that neutralize coagulation FVIII, leading to spontaneous bleeding in soft tissues, mucous membranes, and, in severe cases, life-threatening hemorrhages. It is frequently associated with autoimmune disorders, malignancies, infections, or drug reactions, though in nearly half of cases, no underlying cause is identified.^[8]^ BP, a chronic autoimmune blistering disease of the skin, has been identified as a potential trigger for AHA. BP is caused by autoantibodies against hemidesmosomal proteins (BP180 and BP230), leading to subepidermal blister formation. The association between BP and AHA suggests a common autoimmune mechanism, where immune dysregulation may facilitate the production of FVIII inhibitors.^[9]^ Although the exact pathophysiology remains unclear, immune cross-reactivity, epitope spreading, or polyclonal B-cell activation are proposed mechanisms. Recent studies also suggest that environmental triggers, such as infections or vaccinations, may contribute to AHA development in predisposed individuals.^[10]^

The diagnosis of AHA requires a high index of suspicion, especially in patients with prolonged APTT that does not correct with mixing studies. FVIII activity is markedly reduced, while FVIII inhibitor titers vary significantly. Interestingly, some patients with BP-associated AHA may have undetectable inhibitor levels, making diagnosis challenging. Our case demonstrated a low FVIII activity (6.4%) but normal inhibitor titer, supporting the need for clinical correlation in suspected cases.^[11]^ Several possible explanations have been proposed for this unusual finding. First, the sensitivity of the Bethesda assay may be insufficient to detect low-titer or transient inhibitors, particularly in elderly patients. Second, inhibitors may have been present only temporarily and subsequently cleared before testing. Third, technical factors or dilution effects during the assay could also contribute to false-negative results. Finally, accelerated consumption of FVIII in the setting of active bleeding may result in markedly reduced FVIII activity even in the absence of a detectable inhibitor. These considerations highlight the importance of integrating clinical presentation, laboratory findings, and treatment response when diagnosing BP-associated AHA. In elderly patients, immunosuppressive therapy must be carefully tailored due to increased risks of infections and organ dysfunction. Our patient achieved complete remission with corticosteroid monotherapy, highlighting that aggressive immunosuppression may not always be necessary in frail individuals.^[12]^ Most patients respond within 3 to 6 weeks, with recurrence occurring in 20% to 30% of cases, necessitating long-term follow-up.^[13]^

The association between BP and AHA has been documented in several case studies, highlighting a potential autoimmune link between these 2 conditions. Epidemiologically, acquired hemophilia A is a rare condition with an annual incidence estimated at approximately 1 to 1.5 cases per million individuals.^[1]^ The occurrence of AHA in patients with bullous pemphigoid is exceptionally uncommon and has been reported almost exclusively in isolated case reports and small case series. This rarity highlights the significance of our case, further emphasizing the need for clinicians to remain vigilant for AHA when BP patients present with unexplained bleeding and prolonged APTT. Similar to our case, multiple reports describe elderly patients with BP who develop spontaneous bleeding and prolonged APTT, ultimately diagnosed with AHA. For instance, Binet et al reported a case of BP-associated AHA successfully managed with corticosteroids, demonstrating that immunosuppressive therapy alone can be effective in some patients without requiring additional cytotoxic agents.^[8]^ Another case by Fakprapai and Wattanakrai detailed a patient with severe BP-associated AHA who presented with life-threatening hemorrhages, requiring factor replacement therapy alongside immunosuppression, similar to our patient’s need for aPCC for gastrointestinal bleeding.^[12]^ While most cases emphasize corticosteroid-based treatment, some reports suggest that rituximab or cyclophosphamide may be required in steroid-resistant cases.^[13]^ Interestingly, in contrast to our patient, a study by Qiu et al reported a case of BP-associated AHA where FVIII inhibitor levels were significantly elevated, reinforcing that FVIII inhibitor titers can vary widely between cases, making diagnosis challenging.^[11]^

The prognosis of AHA varies depending on underlying conditions, bleeding severity, and treatment response. While mortality rates of untreated AHA can be as high as 20% to 30%, early recognition and individualized therapy improve survival rates. Our case underscores the importance of early diagnosis, careful selection of immunosuppressive therapy, and close monitoring to prevent life-threatening complications. The association between BP and AHA further emphasizes the need for dermatologists and hematologists to collaborate in evaluating autoimmune-related coagulopathies.^[14]^

In conclusion, this case highlights the rare but significant association between bullous pemphigoid and acquired hemophilia A. Clinicians should remain vigilant for AHA in patients with autoimmune skin diseases presenting with spontaneous bleeding and prolonged APTT. Prompt diagnosis and tailored therapy can lead to favorable outcomes, even in elderly patients with multiple comorbidities.

## Author contributions

**Conceptualization:** Junzhu Xu.

**Data curation:** Zhongmei Liu.

**Formal analysis:** Zhongmei Liu, Junzhu Xu.

**Investigation:** Zhongmei Liu.

**Methodology:** Zhongmei Liu.

**Writing – original draft:** Zhongmei Liu.

**Writing – review & editing:** Junzhu Xu.
